# Statement of Retraction: Repressing phosphatidylinositol-4,5-bisphosphate 3-kinase catalytic subunit gamma by microRNA-142-3p restrains the progression of hepatocellular carcinoma

**DOI:** 10.1080/21655979.2024.2299565

**Published:** 2024-02-20

We, the Editors and Publisher of the journal *Bioengineered*, have retracted the following article:

Chuanli Zeng, Gang Yuan, Yaoren Hu, Donghui Wang, Xiaojun Shi, Dedong Zhu, Airong Hu, Yina Meng & Jialin Lu (2022) Repressing phosphatidylinositol-4,5-bisphosphate 3-kinase catalytic subunit gamma by microRNA-142-3p restrains the progression of hepatocellular carcinoma, Bioengineered, 13:1, 1491-1506, DOI: 10.1080/21655979.2021.1996315

Since publication, concerns have been raised about the integrity of the data in the article. When approached for an explanation, the authors have been unable to fully address the concerns raised and have not been able to provide their original data in the appropriate format. As verifying the validity of published work is core to the integrity of the scholarly record, we are therefore retracting the article. All authors listed in this publication have been informed.

We have been informed in our decision-making by our policy on publishing ethics and integrity and the COPE guidelines on retractions.

The retracted article will remain online to maintain the scholarly record, but it will be digitally watermarked on each page as ‘Retracted’.

